# Association of Tumor Treating Fields (TTFields) therapy with survival in newly diagnosed glioblastoma: a systematic review and meta-analysis

**DOI:** 10.1007/s11060-023-04348-w

**Published:** 2023-07-26

**Authors:** Matthew T. Ballo, Patrick Conlon, Gitit Lavy-Shahaf, Adrian Kinzel, Josef Vymazal, Aaron M. Rulseh

**Affiliations:** 1 Department of Radiation Oncology, West Cancer Center, Germantown, TN USA; 2 Novocure Inc, New York, NY USA; 3 Novocure Ltd, Haifa, Israel; 4 Novocure GmBH, Munich, Germany; 5 Na Homolce Hospital, Prague, Czech Republic

**Keywords:** Glioblastoma, Tumor Treating Fields, Overall survival, TTFields usage, Real-world, Meta-analysis

## Abstract

**Purpose:**

Tumor Treating Fields (TTFields) therapy, an electric field-based cancer treatment, became FDA-approved for patients with newly diagnosed glioblastoma (GBM) in 2015 based on the randomized controlled EF-14 study. Subsequent approvals worldwide and increased adoption over time have raised the question of whether a consistent survival benefit has been observed in the real-world setting, and whether device usage has played a role.

**Methods:**

We conducted a literature search to identify clinical studies evaluating overall survival (OS) in TTFields-treated patients. Comparative and single-cohort studies were analyzed. Survival curves were pooled using a distribution-free random-effects method.

**Results:**

Among nine studies, seven (N = 1430 patients) compared the addition of TTFields therapy to standard of care (SOC) chemoradiotherapy versus SOC alone and were included in a pooled analysis for OS. Meta-analysis of comparative studies indicated a significant improvement in OS for patients receiving TTFields and SOC versus SOC alone (HR: 0.63; 95% CI 0.53–0.75; *p* < 0.001). Among real-world post-approval studies, the pooled median OS was 22.6 months (95% CI 17.6–41.2) for TTFields-treated patients, and 17.4 months (95% CI 14.4–21.6) for those not receiving TTFields. Rates of gross total resection were generally higher in the real-world setting, irrespective of TTFields use. Furthermore, for patients included in studies reporting data on device usage (N = 1015), an average usage rate of ≥ 75% was consistently associated with prolonged survival (*p* < 0.001).

**Conclusions:**

Meta-analysis of comparative TTFields studies suggests survival may be improved with the addition of TTFields to SOC for patients with newly diagnosed GBM.

**Supplementary Information:**

The online version contains supplementary material available at 10.1007/s11060-023-04348-w.

## Introduction

Despite tremendous research focus over the past two decades and advancements in our understanding of the disease, most patients with glioblastoma (GBM) continue to face a poor prognosis, with 5-year survival historically at ~ 5% and virtually all patients experiencing tumor recurrence following initial treatment [1–4]. Multimodal treatment is intensive and has traditionally consisted of maximal safe resection followed by concurrent chemoradiotherapy and maintenance chemotherapy with temozolomide (TMZ) (regimen described by Stupp et al.) [5]. In addition to age and extent of resection being prognostic, *MGMT* promoter methylation has been shown to associate with better response to treatment with alkylating agents like TMZ [6], although only a minority of GBM tumors have this molecular characteristic [7, 8]. Unfortunately, many of the therapy classes that show efficacy in non-central nervous system cancers have failed to show benefit in GBM, underscoring the profound difficulty in developing new therapies for these patients [9].

In 2015, Tumor Treating Fields (TTFields) therapy became an FDA-approved treatment for patients with newly diagnosed (nd) GBM, on the basis of the EF-14 clinical study (NCT00916409) that showed significant extension of progression-free and overall survival (OS) when TTFields therapy was added to maintenance TMZ [1]. TTFields are alternating electric fields that exert physical forces on cancer cells, and work by disrupting processes in the cell that are critical for cancer cell viability and tumor progression [10–12]. TTFields therapy is delivered noninvasively and locoregionally to the tumor through a portable device with arrays placed on the surface of the skin [1]. Preclinical evidence generated across various tumor cell lines and animal models show TTFields to have a selectively cytotoxic effect on cancer cells—synergism with other cancer therapies and downstream immune-stimulating effects have also been observed [13–19]. In patients with newly diagnosed GBM, the addition of TTFields therapy to maintenance TMZ resulted in a median OS of 20.9 months compared with 16.0 months for TMZ alone, with survival improved regardless of age, extent of resection, or *MGMT* methylation status [1]. The therapy was well tolerated, with mild-to-moderate skin irritation from the arrays observed in approximately half of patients, and no systemic toxicities attributed to the treatment [1]. Because the antitumor effects of TTFields therapy are dose-responsive, higher levels of device usage and higher electric field intensity (average intensity through the tumor bed) were each associated with improved survival, independent of other factors [20, 21].

Following approval in the US, additional regulatory approvals and guideline adoptions in Europe and Asia in recent years have expanded awareness of TTFields therapy to broader patient populations and treatment centers. At the same time, preclinical and clinical advancements have added clarity to the therapy’s biological mechanisms of action and have helped further optimize treatment planning [22–26]. Notwithstanding this progress, criticism related to the perceived burdens of using the device and selection biases limiting the generalizability of EF-14 have persisted. Given the direct roles that patients and clinicians each play in applying treatment effectively, it is reasonable to expect a certain degree of variation in patient outcomes between the real world and clinical trial settings, and thus understanding the real-world performance of TTFields therapy has gained increasing focus. Utilizing a systematic review and meta-analytic framework, we therefore sought to assess whether the addition of TTFields to standard of care (SOC) is associated with prolonged survival for patients with newly diagnosed GBM, and whether greater usage of the device translates to benefit in the clinical practice setting.

## Materials and methods

### Systematic review

A systematic literature review was conducted querying PubMed, Embase, and the Cochrane Library to broadly capture literature on clinical studies evaluating OS in patients with GBM treated with TTFields therapy. The search terms *glioblastoma*, *TTFields* OR *tumor treating fields*, and *survival* were utilized along with their synonyms. Literature review and data extraction was performed in accordance with the PRIMSA statement (2020). Studies published in the past 10 years were eligible and collected until January 12, 2023. Studies were stratified into two groups based on the analysis of interest. The first analysis included studies evaluating survival in adults with newly diagnosed GBM treated with TTFields and standard chemoradiotherapy (TMZ-based standard chemoradiotherapy following maximal surgical resection as per the Stupp et al. protocol). Studies that included TTFields and non-TTFields treatment groups were further selected for quantitative assessment. Studies of newly diagnosed patients evaluating the concomitant use of TTFields and investigational therapies were excluded. For the second analysis, studies of patients with newly diagnosed  or recurrent GBM were included if there was an evaluation of survival by the level of TTFields device usage. All studies required objective data on hazard ratio (HR) for OS or Kaplan–Meier (KM) data to be reported. Articles were excluded if there was > 15% overlap with patients included in another study or if they did not provide appropriate outcome data for analysis.

### Data extraction

Prognostic data describing patient, tumor, and treatment characteristics were extracted for each study, or estimated where sufficient information was available. Collected data included age, sex, performance status, *IDH1* status, *MGMT* promoter methylation status, extent of resection, and TTFields device usage. As an estimate of treatment effect, the outcome of interest was HR for OS and corresponding 95% CIs. In cases where HR information was not provided in the text, KM data were used to estimate HR according to the method described by Tierney et al., or were provided by authors directly [27]. For the study by Ballo et al., KM data for the all-comer TTFields group was provided by M.T.B. [28]. Other extracted endpoints included median OS, 2- and 4-year survival rates, and median OS for select patient subgroups where reported.

### Quality assessment

Study quality was assessed using the Newcastle–Ottawa Scale, which is a tool for evaluating the methodological quality of nonrandomized studies (Supporting Table S1). Rating categories include cohort selection, comparability, and outcome reporting, with higher scores indicating higher quality and lower risk of bias [29]. Quality assessment was performed by two reviewers.

### Statistical analysis

Pooled-effect analyses were conducted using STATA 17.0 software (StataCorp, College Station, TX, USA). A random-effects model was used to assess pooled HRs, with the DerSimonian-Laird estimation method applied. Inter-study heterogeneity in effect estimates was evaluated using the Cochran Q (chi-squared) test and the I^2^ statistic. Heterogeneity level was considered moderate if I^2^ values were > 25% [30, 31]. To examine the impact of individual studies on overall effect, sensitivity analyses were performed using a sequential study elimination approach and carried out with RevMan v5.4. To assess median OS and survival rate endpoints, pooled analysis of KM curves for the comparative studies was conducted following a method described by Combescure et al. [32]. Published KM curves were digitized, utilizing Digitzelt, and numbers of at-risk patients were extracted at fixed intervals where available, or estimated following methodology proposed by Tierney et al. [27]. The R MetaSurv package was used to estimate pooled median OS, survival rates, and 95% CIs for both TTFields and non-TTFields treatment groups. Pooled survival curves were illustrated. For all analyses, significance was established using 95% CIs or *p* < 0.05.

## Results

### Study identification and patient characteristics

**Fig. 1:**
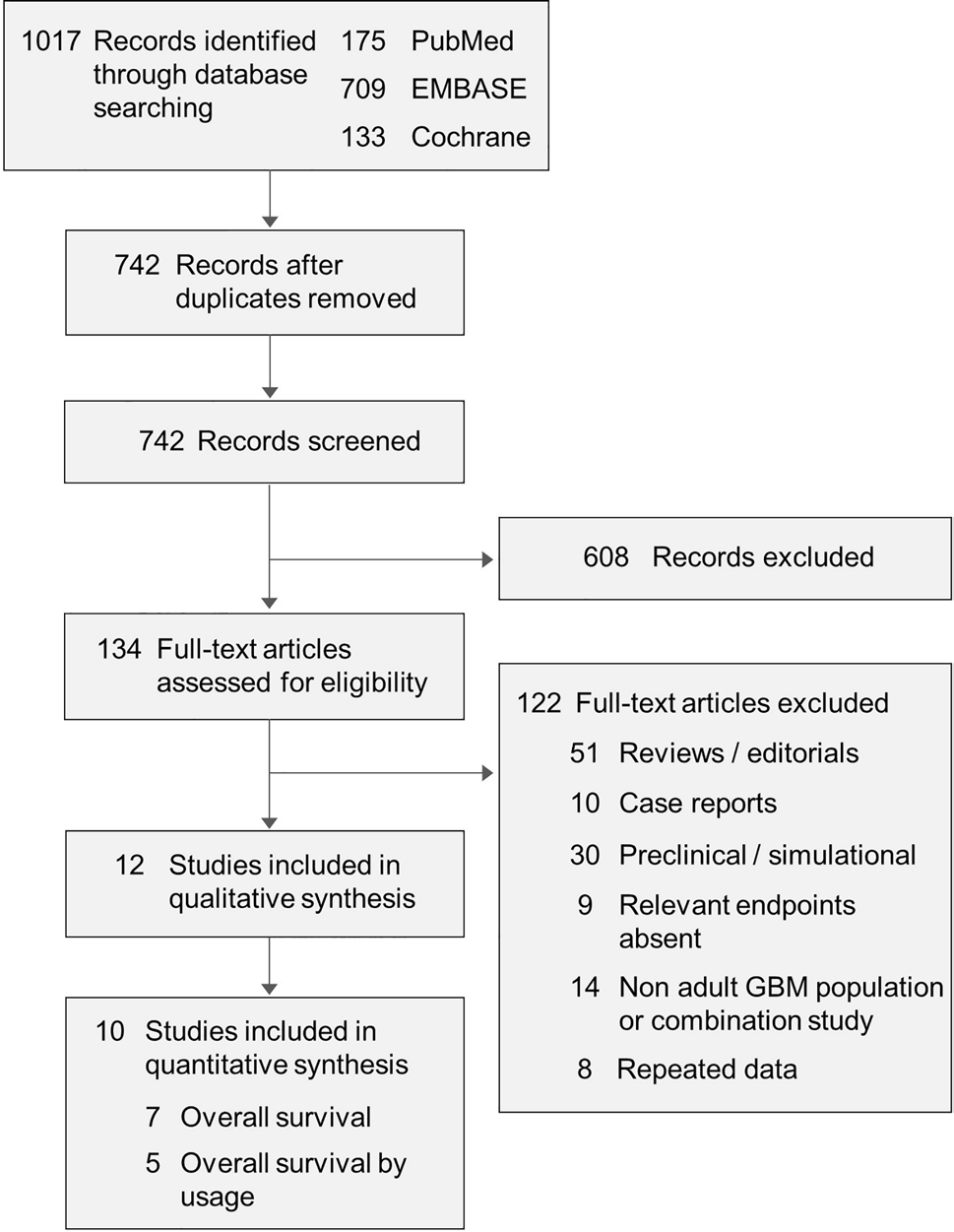
PRISMA flow chart.* GBM* glioblastoma

Following a systematic review of the literature, nine studies were identified that reported on patients treated with TTFields and SOC in the newly diagnosed GBM setting. SOC predominantly included maximal surgical resection and TMZ-based standard chemoradiotherapy according to the Stupp et al. protocol [5]. A PRISMA flow diagram is shown in Fig. 1. Overall, the group consisted of one randomized control trial (695 patients) and eight retrospective cohort studies: two single-cohort studies and six comparative studies (735 patients), in which an intra-study control group of patients not treated with TTFields was used for comparison. Among the cohort studies, three were conducted in the United States, two in Europe, and three in Asia. The six comparative studies included 282 patients treated with TTFields therapy plus SOC, and 453 patients treated with SOC alone. With the exception of 19 patients that were treated with TTFields therapy in earlier clinical trials as reported in the Vymazal study [33], patients in the comparative cohort studies were representative of a real-world dataset. All studies reported OS data.

Study and patient characteristics for the included studies are tabulated in Table 1 for both the TTFields and non-TTFields treatment groups. Median age varied between 48–61 years for the TTFields groups, and 48–65 years for the non-TTFields groups. Maximum age ranged between 63–81 and 75–83 years of age, respectively, for patients in the cohort studies. *IDH1* was mutated in ≤ 10% of evaluable patients across all comparative studies, regardless of TTFields treatment. The ratio of unmethylated-to-methylated *MGMT* promoter status was higher for the TTFields group compared with the non-TTFields group for all but one of the studies. Differences in extent of resection varied 9–13% between the two treatment groups across studies. The proportion of patients with gross total resection (GTR) was > 5% higher for the non-TTFields group compared with the TTFields group in three of the five studies with resection data available and was > 5% lower for the two remaining studies. In total, rates of GTR differed from EF-14 by > 10% in six of the 10 real-world cohorts, and in all six groups, the rate of GTR was higher than the respective group in EF-14.

**Table 1:** Clinical studies evaluating the addition of TTFields therapy to SOC chemoradiotherapy in newly diagnosed glioblastoma

Study	Region	Group	N	Age (range)	Sex		KPS		IDH1		MGMT		Resection		Usage	OS	HR (95% CI)	Additional outcomes
					M	F	Med	≤ 80	mut	WT	M	UNM	GTR	nGTR				
Stupp et al. 2017 (EF-14) [1]	Global	TTFields + SOC	466	56 (19–83)	68	32	90	33	7	92	36	54	53	47	> 75%	20.9	0.63 (0.53–0.76)	2-yr OS: 43% vs. 31% 4-yr OS: 20% vs. 8% Median OS (uMGMT): 16.9 vs. 14.7 mo
		SOC	229	57 (19–80)	69	31	90	32	5	95	42	51	54	46	–	16.0		
Liu et al. 2020 [43]	US	TTFields + SOC	37	61 (28–81)	62	38	90	21.6	8.1	89.2	16.2	62.2	56.8	43.2	NA	15^a^	0.93 (0.58–1.47)	2-yr OS: 21% vs. 28% 4-yr OS: 6% vs. 5%
		SOC	67	65 (28–83)	57	43	90	40.3	4.5	82.1	35.8	35.8	46.3	53.7	–	15^a^		
Chen et al. 2022 [34]	China	TTFields + SOC	63	51	48	52	80	–	8	89	32	60	70	30	87%^b^	21.8	0.43 (0.28–0.67)	2-yr OS: 49% vs. 20% 3-yr OS: 33% vs. 0%,
		SOC	204	54	65	35	90	–	10	88	21	32	79	21	–	15		
Krigers et al. 2022^c^ [44]	Austria	TTFields + SOC	48	57	65	35	–	–	23	67	60	40	–	–	NA	22.6^d^	–	Median OS (uMGMT): 16.7 mo
Ballo et al. 2022 [28]	US	TTFields + SOC	59	59^e^	71	29	–	–	–	–	47.5	44.2	64.5	35.5	57%^f^–84%^g^	20.7^ h^	0.63 (0.38–1.05)	2-yr OS: 34% vs. 24% 4-yr OS: 13% vs. 0%
		SOC	32	63^e^	62.5	37.5	–	–	–	–	47	40.2	53.2	46.8	–	15^ h^		
Pandey et al. 2022 [45]	US	TTFields + SOC	55	59 (26–79)	69	31	–	–	9	–	45	–	–	–	60%	25.5^i^	0.54 (0.31–0.94)	2-yr OS: 56% vs. 35% 3-yr OS: 34% vs. 21%
		SOC	57	58 (17–75)	60	40	–	–	5	–	46	–	–	–	–	18.8^i^		
Nishikawa et al. 2023 [46]	Japan	TTFields + SOC	40	59 (19–75)	62.5	37.5	90	32.5	–	–	–	–	57.5	37.5	> 75%	NR	–	2-yr OS: 53.6%
Vymazal et al. 2023 [33]	Czech Republic	TTFields + SOC	55	48 (22–78)	64	36	80	52.7	7.3	41.8	27.3	12.7	69.1	30.9	82%	31.7	0.61 (0.39–0.95)	2-yr OS: 61% vs. 53% 4-yr OS: 34% vs. 18%
		SOC	54	52 (27–77)	63	37	80	55.6	1.8	42.6	16.7	14.8	79.6	20.4	–	24.8		
She et al. 2023 [47]	China	TTFields + SOC	13	54 (33–63)	54	46	–	30.8^j^	0	100	23.1	76.9	46.2	53.8	91.9^k^	24.8	1.21 (0.45–3.29)	2-yr OS: 54% vs. 38% 4-yr OS: 42% vs. 20%
		SOC	39	48 (22–75)	62	38	–	23.1^j^	0	100	33.3	66.7	59.0	41.0	–	18.6		

Age, usage, and OS columns report data as median values unless otherwise noted; sex, KPS/ECOG, IDH1, MGMT, and resection columns report data as percentages. Where percentage quantities do not total 100, patient data was unavailable or could not be evaluated

*CI* confidence interval; *ECOG* Eastern Cooperative Oncology Group; *GTR* gross total resection; *HR* hazard ratio; *KPS* Karnofsky performance status; *M* methylated; *mut* mutated; *nGTR* not GTR; *OS* overall survival; *SOC* standard of care (regimen described by Stupp et al.); *TTFields* Tumor Treating Fields; *UNM* unmethylated; *WT* wild type

^a^Median survival estimated from Simon and Makuch survival plots; landmark survival rates published

^b^Minimum of 4 weeks for all patients

^c^One patient with recurrent glioblastoma was part of cohort

^d^OS reported as the mean, as opposed to median

^e^Age reported as mean

^f^Patients with < 75% usage or < 2 months duration of use

^g^Patients with ≥ 75% usage and > 2 months duration of use

^h^OS calculated from date of resection or biopsy

^i^OS and progression-free survival were calculated from the date of histological diagnosis

^j^Percentage reflects KPS ≤ 70.

^k^Usage reported as average

### Meta-analysis for overall survival

**Fig. 2:**
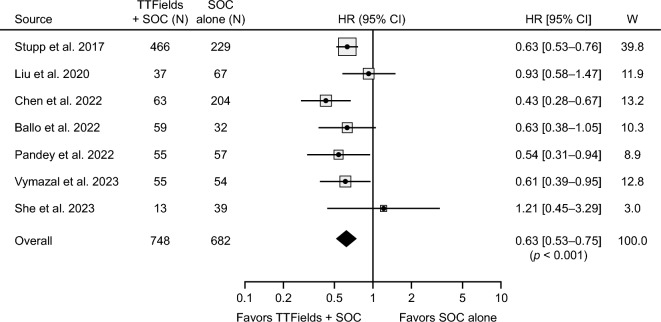
Pooled-effect analysis of overall survival for patients with newly diagnosed glioblastoma treated with TTFields therapy and SOC or SOC alone. The 95% CIs are indicated by horizontal lines. Marker size represents the relative weight of each study as it contributes to the overall pooled effect.* CI* confidence interval;* HR* hazard ratio;* SOC* standard of care;* TTFields* Tumor Treating Fields;* W* weight

Meta-analysis of patients with newly diagnosed GBM revealed significantly improved OS when patients were treated with TTFields therapy and SOC compared with SOC alone (HR: 0.63; 95% CI 0.53–0.75; *p* < 0.001) (Fig. 2). Heterogeneity among studies was low (*I*^2^ = 21%, *p* = 0.27) and a sensitivity analysis indicated that the pooled effect was robust and not dependent on any individual study. A subgroup analysis was conducted to reduce the influence of large datasets and assess survival impact of TTFields in the real-world setting more specifically. In this analysis the Stupp 2017 dataset was removed [1], and a smaller propensity-score matched dataset within the Chen 2022 study was utilized [34]. Meta-analysis of the post-approval studies was consistent with the full dataset in showing a survival benefit with TTFields added to standard chemoradiotherapy (HR: 0.66; 95% CI 0.54–0.82; *p* < 0.001) (Supporting Fig. S1).

**Fig. 3:**
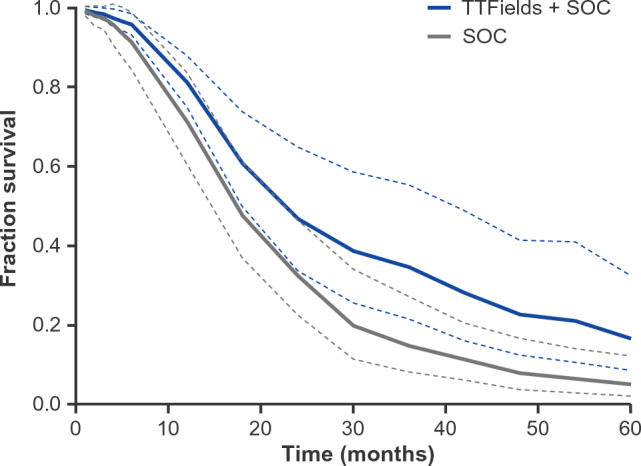
Pooled survival analysis of patients with newly diagnosed glioblastoma treated with TTFields and SOC or SOC alone in the post-approval setting. Pooled Kaplan–Meier (KM) overall survival curves for patients treated with TTFields therapy and SOC (blue) and SOC alone (gray). 95% CIs are represented with dashed lines.* CI* confidence interval;* SOC* standard of care;* TTFields* Tumor Treating Fields

To further assess survival outcomes for patients treated with and without TTFields, survival curves were pooled across comparative studies comprising the real-world dataset (Fig. 3). Among post-approval studies, the pooled median OS was 22.6 months (95% CI 17.6–41.2) for TTFields-treated patients and 17.4 months (95% CI 14.4–21.6) for patients not receiving TTFields. Two-year OS rate was 46.8% (95% CI 33.8–64.8) and 32.3% (95% CI 22.5–46.5) for the TTFields and non-TTFields groups, respectively. Four-year OS rate was 22.7% (95% CI 12.5–41.4) and 8.0% (95% CI 3.8–16.6), respectively.

### Overall survival by TTFields usage

**Fig. 4:**
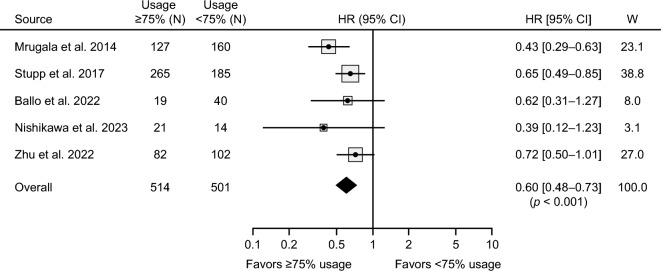
Pooled-effect analysis of overall survival for patients by TTFields device usage rate. Forest plot depicts OS HRs for studies evaluating survival across 75% usage threshold. The 95% CIs are indicated by horizontal lines. Marker size represents the relative weight of each study as it contributes to the overall pooled effect.* CI* confidence interval;* HR* hazard ratio;* TTFields* Tumor Treating Fields;* W* weight

In addition to the assessment of survival in the newly diagnosed setting, six studies were identified in the review that evaluated the impact of device usage rate on patient survival, with five of the six studies utilizing HR as the metric for evaluation. Survival was evaluated across the 75% device usage rate threshold (average of 18 h/day), which most centers have established as the recommended minimum level of device usage for optimal benefit. Patient characteristics and survival outcomes are tabulated in Supporting Table S2. Meta-analysis indicated an improvement in OS when average device usage was ≥ 75% compared with < 75% (HR: 0.60; 95% CI 0.48–0.73; *p* < 0.001; *I*^2^ = 15%) (Fig. 4). In a subgroup analysis of patients treated exclusively in the real-world setting, the survival benefit was maintained with higher device usage (HR: 0.56; 95% CI 0.41–0.76; *p* < 0.001), with inter-study heterogeneity at a low-to-moderate level (*I*^2^ = 28%, *p* = 0.24).

## Discussion

Increases in global approvals and collective experience with TTFields therapy in recent years have given way to a number of institution-led studies and case reports of TTFields use among patients with newly diagnosed GBM, a reflection of clinical uptake and potential utility. However, a clear understanding of how TTFields therapy performs within its approved indications is lacking, and perception of clinical benefit continues to vary across treatment centers. From a comprehensive pooled analysis of comparative studies spanning multiple geographic regions, we found that adding TTFields therapy to standard of care treatment was significantly associated with improved survival for patients with newly diagnosed GBM. Additionally, the magnitude of the survival benefit with TTFields in the real-world setting was shown to be consistent with that of the pivotal EF-14 trial, with an increase in median OS of approximately 5 months, and an overall reduction in risk of death in the 30–40% range versus standard chemoradiotherapy alone.

Patient health and treatment conditions will typically vary more widely for patients treated in clinical practice as opposed to a trial setting, with prognosis often worse for many patients. As expected in this analysis, variability between real-world cohorts and EF-14 was observed across several baseline factors. Interestingly, the rate of GTR was the only factor that consistently differed from the EF-14 study, with rates generally higher for both treatment groups. When comparing between patients treated with and without TTFields therapy, variability was also observed across certain prognostic factors, notably *MGMT* methylation status and resection status, but the direction of variation was mixed with respect to favoring the TTFields group or the non-TTFields group. While patients treated with TTFields appeared to have a slightly younger median age than those who did not receive TTFields (up to 4 years), it was not clear if this translated into meaningful differences in functional status or patient well-being. The median age range of patients treated with TTFields varied between 48 years and 61 years of age, but there was no consistent age difference between patients in the real world and EF-14. This is in line with findings from a recent post-market safety study and suggests that age is not a barrier in a patient’s candidacy for TTFields therapy [35]. Higher rates of GTR, measurement of survival from time of surgery, and general population-level improvements in patient health and care may explain the modest extensions in survival rates when compared with the earlier EF-14 trial. Despite these differences, patients treated with TTFields continued to show an improvement in OS in the real-world setting.

Prior analyses of patients treated with TTFields have shown correlation of OS with both device usage rate and field intensity within the tumor bed [20, 26, 28, 36]. The EF-14 and EF-11 (NCT00379470) studies in patients with newly diagnosed and recurrent GBM, respectively, showed significant survival improvements when TTFields device usage was ≥ 75% compared with < 75% [1, 37]. Based on these findings and the fact that most patients in the trials achieved these thresholds, an average monthly device usage rate of 75% became the recommended target level for patients and is what most treatment centers communicate as part of their education to patients and caregivers. When assessing survival across this threshold in the real-world setting, a consistent survival benefit was observed for patients with usage of ≥ 75%. A previous analysis indicated that patients meeting this usage threshold may also be more likely to sustain treatment with TTFields for a longer period of time, suggesting a link between usage and duration of treatment [28]. Indeed, it has been shown that for patients with recurrent GBM treated with TTFields therapy, time to response is often slow and can take several months in some cases [38], consistent with a potential immune system role. As with usage rate, the length of time patients receive treatment with TTFields may vary in clinical practice, and depends in part on the continuation of TTFields through first tumor progression, as was allowed in the pivotal EF-14 trial. The impact of treatment duration (e.g., length of sustained treatment after treatment start) on survival outcomes is unclear and warrants further investigation.

Beyond the studies identified in this review, additional investigations have highlighted the use of TTFields therapy in subsets of patients with high disease burden, and concomitant with other therapeutic modalities. A small cohort study of patients in the UK with unmethylated *MGMT* status showed a prolongation of 3.3 months with TTFields added to standard therapy [39], although the sample size was small and did not reach statistical significance. Recent meta-analyses of TTFields concomitant with various other therapies have also suggested survival improvements [40–42]. Our analysis builds on a growing body of research evaluating the efficacy of TTFields therapy in GBM, and brings particular focus to the survival impact of adding TTFields to standard of care therapy in newly diagnosed patients.

There are several limitations of this meta-analysis worth noting. As with all non-prospective and non-randomized studies, risks of bias and overestimations of treatment effect can exist. While all studies included in this analysis were retrospective, we did not detect any significant imbalances in patient prognostic factors that were in favor of either treatment group, or that otherwise indicated risk of bias in patient selection or treatment. Overall, patients in the comparative studies were well described in terms of known prognostic factors. Additionally, as TTFields therapy is a relatively new treatment, it remains possible that additional prognostic factors, beyond those routinely captured for GBM patients, may exist, including those related to caregiver/family support and patient socio-economic factors. Regarding treatment-specific factors, patterns related to how long patients sustain treatment with TTFields, as well as sources of variability in treatment duration, remain unknown and will be important to examine. Understanding the role that these factors, as well as additional device- and molecular-based factors, might play in impacting survival will help further guide clinical decision making.

In conclusion, the results of this meta-analysis suggest the addition of TTFields to standard chemoradiotherapy significantly prolongs OS for newly diagnosed patients with GBM treated in the real-world setting. While device usage rate appears to vary in clinical practice, the association of high device usage rate and survival is consistent with patients treated in the pivotal clinical trials, with many patients able to attain high usage. Future studies will be important to investigate the role of TTFields treatment duration in patient outcomes, and further assess clinical benefit in high-unmet need populations, including patients with unmethylated *MGMT* promoter status.

## Supplementary Information

Below is the link to the electronic supplementary material.

Supplementary material 1 (DOCX 180 kb)

## Data Availability

The datasets generated during and/or analyzed during the current study are available on reasonable request.

## References

[1] Stupp R, Taillibert S, Kanner A, Read W, Steinberg D, Lhermitte B, Toms S, Idbaih A, Ahluwalia MS, Fink K, Di Meco F, Lieberman F, Zhu JJ, Stragliotto G, Tran D, Brem S, Hottinger A, Kirson ED, Lavy-Shahaf G, Weinberg U, Kim CY, Paek SH, Nicholas G, Bruna J, Hirte H, Weller M, Palti Y, Hegi ME, Ram Z (2017). Effect of Tumor-Treating Fields Plus maintenance temozolomide vs maintenance temozolomide alone on survival in patients with glioblastoma: a randomized clinical trial. JAMA.

[2] Ostrom QT, Price M, Neff C, Cioffi G, Waite KA, Kruchko C, Barnholtz-Sloan JS (2022). CBTRUS Statistical Report: primary brain and other Central Nervous System Tumors diagnosed in the United States in 2015–2019. Neuro Oncol.

[3] Ostrom QT, Gittleman H, Liao P, Vecchione-Koval T, Wolinsky Y, Kruchko C, Barnholtz-Sloan JS (2017). CBTRUS Statistical Report: primary brain and other central nervous system tumors diagnosed in the United States in 2010–2014. Neuro Oncol.

[4] Birzu C, French P, Caccese M, Cerretti G, Idbaih A, Zagonel V, Lombardi G (2020). Recurrent glioblastoma: from molecular landscape to new treatment perspectives. Cancers.

[5] Stupp R, Mason WP, van den Bent MJ, Weller M, Fisher B, Taphoorn MJ, Belanger K, Brandes AA, Marosi C, Bogdahn U, Curschmann J, Janzer RC, Ludwin SK, Gorlia T, Allgeier A, Lacombe D, Cairncross JG, Eisenhauer E, Mirimanoff RO (2005). Radiotherapy plus concomitant and adjuvant temozolomide for glioblastoma. N Engl J Med.

[6] Rivera AL, Pelloski CE, Gilbert MR, Colman H, De La Cruz C, Sulman EP, Bekele BN, Aldape KD (2010). MGMT promoter methylation is predictive of response to radiotherapy and prognostic in the absence of adjuvant alkylating chemotherapy for glioblastoma. Neuro Oncol.

[7] Hegi ME, Diserens A-C, Gorlia T, Hamou M-F, de Tribolet N, Weller M, Kros JM, Hainfellner JA, Mason W, Mariani L, Bromberg JEC, Hau P, Mirimanoff RO, Cairncross JG, Janzer RC, Stupp R (2005). MGMT gene silencing and benefit from temozolomide in glioblastoma. N Engl J Med.

[8] Gilbert MR, Wang M, Aldape KD, Stupp R, Hegi ME, Jaeckle KA, Armstrong TS, Wefel JS, Won M, Blumenthal DT, Mahajan A, Schultz CJ, Erridge S, Baumert B, Hopkins KI, Tzuk-Shina T, Brown PD, Chakravarti A, Curran WJ, Mehta MP (2013). Dose-dense temozolomide for newly diagnosed glioblastoma: a randomized phase III clinical trial. J Clin Oncol.

[9] Hanif F, Muzaffar K, Perveen K, Malhi SM, Simjee Sh U (2017). Glioblastoma multiforme: a review of its epidemiology and pathogenesis through clinical presentation and treatment. Asian Pac J Cancer Prev.

[10] Mun EJ, Babiker HM, Weinberg U, Kirson ED, Von Hoff DD (2018). Tumor-treating Fields: a fourth modality in cancer treatment. Clin Cancer Res.

[11] Kirson ED, Gurvich Z, Schneiderman R, Dekel E, Itzhaki A, Wasserman Y, Schatzberger R, Palti Y (2004). Disruption of cancer cell replication by alternating electric fields. Cancer Res.

[12] Voloshin T, Schneiderman RS, Volodin A, Shamir RR, Kaynan N, Zeevi E, Koren L, Klein-Goldberg A, Paz R, Giladi M, Bomzon Z, Weinberg U, Palti Y (2020). Tumor Treating Fields (TTFields) hinder cancer cell motility through regulation of microtubule and actin dynamics. Cancers.

[13] Voloshin T, Kaynan N, Davidi S, Porat Y, Shteingauz A, Schneiderman RS, Zeevi E, Munster M, Blat R, Tempel Brami C, Cahal S, Itzhaki A, Giladi M, Kirson ED, Weinberg U, Kinzel A, Palti Y (2020). Tumor-treating fields (TTFields) induce immunogenic cell death resulting in enhanced antitumor efficacy when combined with anti-PD-1 therapy. Cancer Immunol Immunother.

[14] Barsheshet Y, Voloshin T, Brant B, Cohen G, Koren L, Blatt R, Cahal S, Haj Khalil T, Zemer Tov E, Paz R, Klein-Goldberg A, Tempel-Brami C, Jacobovitch S, Volodin A, Kan T, Koltun B, David C, Haber A, Giladi M, Weinberg U, Palti Y (2022). Tumor Treating Fields (TTFields) concomitant with immune checkpoint inhibitors are therapeutically effective in non-small cell lung cancer (NSCLC) in vivo model. Int J Mol Sci.

[15] Giladi M, Weinberg U, Schneiderman RS, Porat Y, Munster M, Voloshin T, Blatt R, Cahal S, Itzhaki A, Onn A, Kirson ED, Palti Y (2014). Alternating electric fields (tumor-treating fields therapy) can improve chemotherapy treatment efficacy in non-small cell lung cancer both *in vitro* and *in vivo*. Semin Oncol.

[16] Giladi M, Schneiderman RS, Porat Y, Munster M, Itzhaki A, Mordechovich D, Cahal S, Kirson ED, Weinberg U, Palti Y (2014). Mitotic disruption and reduced clonogenicity of pancreatic cancer cells in vitro and in vivo by tumor treating fields. Pancreatology.

[17] Voloshin T, Munster M, Blatt R, Shteingauz A, Roberts PC, Schmelz EM, Giladi M, Schneiderman RS, Zeevi E, Porat Y, Bomzon Z, Urman N, Itzhaki A, Cahal S, Kirson ED, Weinberg U, Palti Y (2016). Alternating electric fields (TTFields) in combination with paclitaxel are therapeutically effective against ovarian cancer cells in vitro and in vivo. Int J Cancer.

[18] Mumblat H, Martinez-Conde A, Braten O, Munster M, Dor-On E, Schneiderman RS, Porat Y, Voloshin T, Davidi S, Blatt R, Shteingauz A, Tempel Brami C, Zeevi E, Lajterer C, Shmueli Y, Danilov S, Haber A, Giladi M, Weinberg U, Kinzel A, Palti Y (2021). Tumor Treating Fields (TTFields) downregulate the Fanconi Anemia-BRCA pathway and increase the efficacy of chemotherapy in malignant pleural mesothelioma preclinical models. Lung Cancer.

[19] Kessler AF, Frömbling GE, Gross F, Hahn M, Dzokou W, Ernestus RI, Löhr M, Hagemann C (2018). Effects of tumor treating fields (TTFields) on glioblastoma cells are augmented by mitotic checkpoint inhibition. Cell Death Discov.

[20] Toms SA, Kim CY, Nicholas G, Ram Z (2019). Increased compliance with tumor treating fields therapy is prognostic for improved survival in the treatment of glioblastoma: a subgroup analysis of the EF-14 phase III trial. J Neurooncol.

[21] Ballo MT, Urman N, Lavy-Shahaf G, Grewal J, Bomzon Z, Toms S (2019). Correlation of tumor treating fields dosimetry to survival outcomes in newly diagnosed glioblastoma: a large-scale numerical simulation-based analysis of data from the phase 3 EF-14 randomized trial. Int J Radiat Oncol Biol Phys.

[22] Moser JC, Salvador E, Deniz K, Swanson K, Tusynski J, Carlson KW, Karanam NK, Patel CB, Story M, Lou E, Hagemann C (2022). The mechanisms of action of Tumor Treating Fields. Cancer Res.

[23] Chen D, Le SB, Hutchinson TE, Calinescu AA, Sebastian M, Jin D, Liu T, Ghiaseddin A, Rahman M, Tran DD (2022). Tumor Treating Fields dually activate STING and AIM2 inflammasomes to induce adjuvant immunity in glioblastoma. J Clin Invest.

[24] Lacouture M, Anadkat MJ, Ballo MT, Iwamoto F, Jeyapalan SA, La Rocca RV, Schwartz M, Serventi JN, Glas M (2020). Prevention and management of dermatologic adverse events associated with Tumor Treating Fields in patients with glioblastoma. Front Oncol.

[25] Trusheim J, Dunbar E, Battiste J, Iwamoto F, Mohile N, Damek D, Bota DA, Connelly J (2017). A state-of-the-art review and guidelines for tumor treating fields treatment planning and patient follow-up in glioblastoma. CNS Oncol.

[26] Glas M, Ballo MT, Bomzon Z, Urman N, Levi S, Lavy-Shahaf G, Jeyapalan S, Sio TT, DeRose PM, Misch M, Taillibert S, Ram Z, Hottinger AF, Easaw J, Kim CY, Mohan S, Stupp R (2022). The impact of Tumor Treating Fields on glioblastoma progression patterns. Int J Radiat Oncol Biol Phys.

[27] Tierney JF, Stewart LA, Ghersi D, Burdett S, Sydes MR (2007). Practical methods for incorporating summary time-to-event data into meta-analysis. Trials.

[28] Ballo MT, Qualls KW, Michael LM, Sorenson JM, Baughman B, Karri-Wellikoff S, Pandey M (2022). Determinants of tumor treating field usage in patients with primary glioblastoma: a single institutional experience. Neurooncol Adv.

[29] Wells G, Shea B, O’Connell D, Peterson j, Welch V, Losos M, Tugwell P (2000) The Newcastle–Ottawa Scale (NOS) for Assessing the Quality of Non-Randomized Studies in Meta-Analysis. https://www.ohri.ca/programs/clinical_epidemiology/oxford.asp. Accessed 13 Mar 2023

[30] Higgins JP, Thompson SG, Deeks JJ, Altman DG (2003). Measuring inconsistency in meta-analyses. BMJ.

[31] West SL, Gartlehner G, Mansfield AJ, Poole C, Tant E, Lenfestey N, Lux LJ, Amoozegar J, Morton SC, Carey TC, Viswanathan M, Lohr KN (2010) AHRQ methods for Effective Health Care. Comparative effectiveness review methods: clinical heterogeneity. Agency for Healthcare Research and Quality (US), Rockville (MD) 21433337

[32] Combescure C, Foucher Y, Jackson D (2014). Meta-analysis of single-arm survival studies: a distribution-free approach for estimating summary survival curves with random effects. Stat Med.

[33] Vymazal J, Kazda T, Novak T, Slanina P, Sroubek J, Klener J, Hrbac T, Syrucek M, Rulseh AM (2023). Eighteen years’ experience with tumor treating fields in the treatment of newly diagnosed glioblastoma. Front Oncol.

[34] Chen C, Xu H, Song K, Zhang Y, Zhang J, Wang Y, Sheng X, Chen L, Qin Z (2022). Tumor treating Fields combine with temozolomide for newly diagnosed glioblastoma: a retrospective analysis of chinese patients in a single center. J Clin Med.

[35] Shi W, Blumenthal DT, Oberheim Bush NA, Kebir S, Lukas RV, Muragaki Y, Zhu JJ, Glas M (2020). Global post-marketing safety surveillance of Tumor Treating Fields (TTFields) in patients with high-grade glioma in clinical practice. J Neurooncol.

[36] Korshoej AR, Lukacova S, Lassen-Ramshad Y, Rahbek C, Severinsen KE, Guldberg TL, Mikic N, Jensen MH, Cortnum SOS, von Oettingen G, Sørensen JCH (2020). OptimalTTF-1: enhancing tumor treating fields therapy with skull remodeling surgery. A clinical phase I trial in adult recurrent glioblastoma. Neurooncol Adv.

[37] Kanner AA, Wong ET, Villano JL, Ram Z (2014). Post hoc analyses of intention-to-treat population in phase III comparison of NovoTTF-100A™ system versus best physician’s choice chemotherapy. Semin Oncol.

[38] Vymazal J, Wong ET (2014). Response patterns of recurrent glioblastomas treated with tumor-treating fields. Semin Oncol.

[39] Olubajo F, Thorpe A, Davis C, Sinha R, Crofton A, Mills SJ, Williams M, Jenkinson MD, Price SJ, Watts C, Brodbelt AR (2022). Tumour treating fields in glioblastoma: is the treatment tolerable, effective, and practical in UK patients?. Br J Neurosurg.

[40] Magouliotis DE, Asprodini EK, Svokos KA, Tasiopoulou VS, Svokos AA, Toms SA (2018). Tumor-treating fields as a fourth treating modality for glioblastoma: a meta-analysis. Acta Neurochir (Wien).

[41] Shah PP, White T, Khalafallah AM, Romo CG, Price C, Mukherjee D (2020). A systematic review of tumor treating fields therapy for high-grade gliomas. J Neurooncol.

[42] Regev O, Merkin V, Blumenthal DT, Melamed I, Kaisman-Elbaz T (2021). Tumor-treating Fields for the treatment of glioblastoma: a systematic review and meta-analysis. Neurooncol Pract.

[43] Liu Y, Strawderman MS, Warren KT, Richardson M, Serventi JN, Mohile NA, Milano MT, Walter KA (2020). Clinical efficacy of Tumor Treating Fields for newly diagnosed glioblastoma. Anticancer Res.

[44] Krigers A, Pinggera D, Demetz M, Kornberger LM, Kerschbaumer J, Thomé C, Freyschlag CF (2022). The routine application of Tumor-Treating Fields in the treatment of glioblastoma WHO° IV. Front Neurol.

[45] Pandey M, Xiu J, Mittal S, Zeng J, Saul M, Kesari S, Azadi A, Newton H, Deniz K, Ladner K, Sumrall A, Korn WM, Lou E (2022). Molecular alterations associated with improved outcome in patients with glioblastoma treated with Tumor-Treating Fields. Neurooncol Adv.

[46] Nishikawa R, Yamasaki F, Arakawa Y, Muragaki Y, Narita Y, Tanaka S, Yamaguchi S, Mukasa A, Kanamori M (2023). Safety and efficacy of tumour-treating fields (TTFields) therapy for newly diagnosed glioblastoma in japanese patients using the Novo-TTF System: a prospective post-approval study. Jpn J Clin Oncol.

[47] She L, Gong X, Su L, Liu C (2022). Effectiveness and safety of tumor-treating fields therapy for glioblastoma: a single-center study in a chinese cohort. Front Neurol.

