# Supplementary material for: Association of Tumor Treating Fields (TTFields) therapy with survival in newly diagnosed glioblastoma: a systematic review and meta-analysis
Source: J Neurooncol. 2023 Jul 26;164(1):1–9. doi: 10.1007/s11060-023-04348-w (PMC10462574; doi:10.1007/s11060-023-04348-w)
Supplement: Supplementary file 1 — Supplementary material 1 (DOCX 180 kb) [file 11060_2023_4348_MOESM1_ESM.docx]

# Supplementary materials

Table S1 Quality assessment according to Newcastle-Ottawa Scale for non-randomized clinical studies

| **Study** | **Selection** | **Comparability** | **Outcome** | **Overall** |
| --- | --- | --- | --- | --- |
| Mrugala et al. 2014 [1] | ** | – | *** | 5 |
| Liu et al. 2020 [2] | *** | * | ** | 6 |
| Chen et al. 2022 [3] | *** | * | *** | 7 |
| Krigers et al. 2022 [4] | ** | - | * | 3 |
| Zhu et al. 2022 [5] | *** | - | *** | 6 |
| Ballo et al. 2022 [6] | *** | * | *** | 7 |
| Pandey et al. 2022 [7] | *** | * | ** | 6 |
| Nishikawa et al. 2023 [8] | ** | - | *** | 5 |
| Vymazal et al. 2023 [9] | *** | * | *** | 7 |
| She et al. 2023 [10] | *** | * | *** | 7 |

Quality guidance:
Good (3 or 4 stars in selection domain AND 1 or 2 stars in comparability domain AND 2 or 3 stars in outcome/exposure domain). Fair (2 stars in selection domain AND 1 or 2 stars in comparability domain AND 2 or 3 stars in outcome/exposure domain).
Poor (0 or 1 star in selection domain OR 0 stars in comparability domain OR 0 or 1 stars in outcome/exposure domain).

Table S2 Clinical studies evaluating survival outcomes for patients with TTFields therapy device usage above and below the 75% threshold

| **Study** | **Setting** | **Region** | **Group** | **N** | **Age** | **Sex** | | **Usage** | **DoT** | **OS** | **Median OS (≥ 75% vs < 75%)** |
| --- | --- | --- | --- | --- | --- | --- | --- | --- | --- | --- | --- |
|  |  |  |  |  |  | **M** | **F** |  |  |  |  |
| Stupp et al 2017 (EF-14) [11] | ndGBM | Global | TTFields + SOC | 466 | 56 | 68 | 32 | >75%^a^ | 8.2 | 20.9 | 22.6 vs 19.1 *p* = 0.009 |
| Ballo et al 2022 [6] | ndGBM | US | TTFields + SOC | 59 | 59 | 71 | 29 | 57%^b^–84%^c^ | 3^b^–9^c^ | 20.7 | 28.0^b^ vs 20.0^c^ |
| Nishikawa et al 2023 [8] | ndGBM | Japan | TTFields + SOC | 40 | 59 | 62.5 | 37.5 | >75%^d^ | – | NR | NR vs 18.8 *p* = 0.135 |
| Stupp et al 2012 (EF-11)^e^ [12] | rGBM | Global | TTFields | 120 | 54 | 77 | 23 | 86%^f^ | 2.3 | 6.6 | 7.7 vs 4.5 *p* = 0.042 |
| Mrugala et al 2014 [1] | rGBM | US | TTFields | 457 | 55 | 68 | 32 | 70%^g^ | 4.1 | 9.6 | 13.5 vs 4.0 *p* < 0.0001 |
| Zhu et al 2022 [5] | rGBM | US | TTFields | 192 | 57 | 65 | 35 | <75%^h^ | – | 7.4 | 9.8 vs 6.7 *p* = 0.059 |

^a^From 450 evaluable patients.
^b^Patients with <75% usage or <2 months duration of use.
^c^Patients with ≥75% usage and >2 months duration of use.
^d^From 36 evaluable patients.
^e^Data from Stupp et al, 2012, and Kanner et al, 2014.
^f^From 116 evaluable patients.
^g^From 287 evaluable patients.
^h^From 184 evaluable patients.
*DoT* directly observed therapy; *GBM* glioblastoma; *ndGBM* newly diagnosed GBM, *NR* not reported; *OS* overall survival; *rGBM*, recurrent GBM; *SOC* standard of care (regimen described by Stupp et al).

Fig. S1 Pooled-effect analysis of overall survival for patients with newly diagnosed glioblastoma treated with TTFields therapy and SOC or SOC alone in the real-world setting. Forest plot depicts overall survival hazard ratios for comparative post-approval studies. The 95% CIs are indicated by horizontal lines. Marker size represents the relative weight of each study as it contributes to the overall pooled effect.


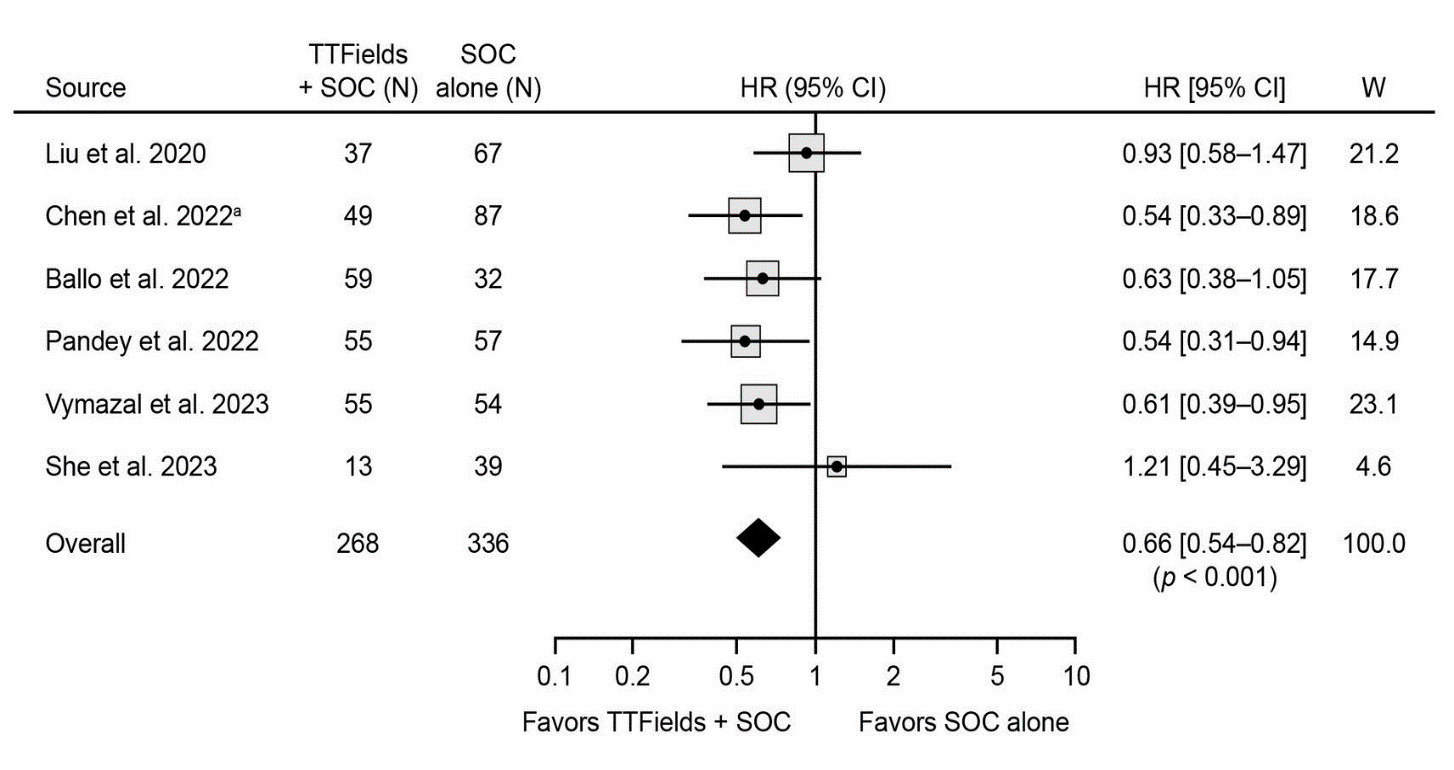


^a^A propensity score-matched dataset in the Chen et al study was utilized.
*CI* confidence interval; *HR* hazard ratio; *TTFields* Tumor Treating Fields; *W* weight.

1. Mrugala MM, Engelhard HH, Dinh Tran D, Kew Y, Cavaliere R, Villano JL, Annenelie Bota D, Rudnick J, Love Sumrall A, Zhu J-J, Butowski N (2014) Clinical practice experience with NovoTTF-100A™ system for glioblastoma: the Patient Registry Dataset (PRiDe). Semin Oncol 41: S4–S13 doi:10.1053/j.seminoncol.2014.09.010

2. Liu Y, Strawderman MS, Warren KT, Richardson M, Serventi JN, Mohile NA, Milano MT, Walter KA (2020) Clinical efficacy of Tumor Treating Fields for newly diagnosed glioblastoma. Anticancer Res 40: 5801–5806 doi:10.21873/anticanres.14597

3. Chen C, Xu H, Song K, Zhang Y, Zhang J, Wang Y, Sheng X, Chen L, Qin Z (2022) Tumor Treating Fields combine with temozolomide for newly diagnosed glioblastoma: a retrospective analysis of Chinese patients in a single center. J Clin Med 11 doi:10.3390/jcm11195855

4. Krigers A, Pinggera D, Demetz M, Kornberger LM, Kerschbaumer J, Thomé C, Freyschlag CF (2022) The routine application of Tumor-Treating Fields in the treatment of glioblastoma WHO° IV. Front Neurol 13: 900377 doi:10.3389/fneur.2022.900377

5. Zhu JJ, Goldlust SA, Kleinberg LR, Honnorat J, Oberheim Bush NA, Ram Z (2022) Tumor Treating Fields (TTFields) therapy vs physicians' choice standard-of-care treatment in patients with recurrent glioblastoma: a post-approval registry study (EF-19). Discov Oncol 13: 105 doi:10.1007/s12672-022-00555-5

6. Ballo MT, Qualls KW, Michael LM, Sorenson JM, Baughman B, Karri-Wellikoff S, Pandey M (2022) Determinants of tumor treating field usage in patients with primary glioblastoma: A single institutional experience. Neurooncol Adv 4: vdac150 doi:10.1093/noajnl/vdac150

7. Pandey M, Xiu J, Mittal S, Zeng J, Saul M, Kesari S, Azadi A, Newton H, Deniz K, Ladner K, Sumrall A, Korn WM, Lou E (2022) Molecular alterations associated with improved outcome in patients with glioblastoma treated with Tumor-Treating Fields. Neurooncol Adv 4: vdac096 doi:10.1093/noajnl/vdac096

8. Nishikawa R, Yamasaki F, Arakawa Y, Muragaki Y, Narita Y, Tanaka S, Yamaguchi S, Mukasa A, Kanamori M (2023) Safety and efficacy of tumour-treating fields (TTFields) therapy for newly diagnosed glioblastoma in Japanese patients using the Novo-TTF System: a prospective post-approval study. Jpn J Clin Oncol: hyad001. doi: 010.1093/jjco/hyad1001 doi:10.1093/jjco/hyad001

9. Vymazal J, Kazda T, Novak T, Slanina P, Sroubek J, Klener J, Hrbac T, Syrucek M, Rulseh AM (2023) Eighteen years’ experience with tumor treating fields in the treatment of newly diagnosed glioblastoma. Front Oncol 12: 1014455 doi:10.3389/fonc.2022.1014455

10. She L, Gong X, Su L, Liu C (2022) Effectiveness and safety of tumor-treating fields therapy for glioblastoma: A single-center study in a Chinese cohort. Front Neurol 13: 1042888 doi:10.3389/fneur.2022.1042888

11. Stupp R, Taillibert S, Kanner A, Read W, Steinberg D, Lhermitte B, Toms S, Idbaih A, Ahluwalia MS, Fink K, Di Meco F, Lieberman F, Zhu JJ, Stragliotto G, Tran D, Brem S, Hottinger A, Kirson ED, Lavy-Shahaf G, Weinberg U, Kim CY, Paek SH, Nicholas G, Bruna J, Hirte H, Weller M, Palti Y, Hegi ME, Ram Z (2017) Effect of Tumor-Treating Fields Plus Maintenance Temozolomide vs Maintenance Temozolomide Alone on Survival in Patients With Glioblastoma: A Randomized Clinical Trial. JAMA 318: 2306-2316 doi:10.1001/jama.2017.18718

12. Stupp R, Wong ET, Kanner AA, Steinberg D, Engelhard H, Heidecke V, Kirson ED, Taillibert S, Liebermann F, Dbaly V, Ram Z, Villano JL, Rainov N, Weinberg U, Schiff D, Kunschner L, Raizer J, Honnorat J, Sloan A, Malkin M, Landolfi JC, Payer F, Mehdorn M, Weil RJ, Pannullo SC, Westphal M, Smrcka M, Chin L, Kostron H, Hofer S, Bruce J, Cosgrove R, Paleologous N, Palti Y, Gutin PH (2012) NovoTTF-100A versus physician's choice chemotherapy in recurrent glioblastoma: a randomised phase III trial of a novel treatment modality. Eur J Cancer 48: 2192–2202 doi:10.1016/j.ejca.2012.04.011
